# Post-Neoadjuvant Treatment in HER2-Positive Breast Cancer: Escalation and De-Escalation Strategies

**DOI:** 10.3390/cancers14123002

**Published:** 2022-06-18

**Authors:** Natalia Krawczyk, Tanja Fehm, Eugen Ruckhaeberle, Laura Brus, Valeria Kopperschmidt, Achim Rody, Lars Hanker, Maggie Banys-Paluchowski

**Affiliations:** 1 Department of Gynecology and Obstetrics, Henrich Heine University Düsseldorf, 40225 Düsseldorf, Germany; tanja.fehm@med.uni-duesseldorf.de (T.F.); eugen.ruckhaeberle@med.uni-duesseldorf.de (E.R.); 2 Regioklinikum Pinneberg, 25421 Pinneberg, Germany; ls.brus@gmail.com (L.B.); sophie.kopperschmidt@student.uni-luebeck.de (V.K.); 3 Department of Gynecology and Obstetrics, University Hospital Schleswig-Holstein Campus Lübeck, 23562 Lübeck, Germany; achim.rody@uksh.de (A.R.); lars.hanker@uksh.de (L.H.); maggie.banys-paluchowski@uksh.de (M.B.-P.)

**Keywords:** breast cancer, post-neoadjuvant therapy, HER2 positive, therapy response, survival

## Abstract

**Simple Summary:**

The response to neoadjuvant treatment is strongly associated with the clinical outcome of breast cancer patients, especially in the HER2-positive subtype of the disease. In HER2-positive patients with a residual tumor burden, an escalation of post-neoadjuvant therapy leads to the improvement of survival, while (post)-neoadjuvant treatment de-escalation is currently being discussed in low-risk settings in order to avoid unnecessary toxicities.

**Abstract:**

Patients with high-risk non-metastatic breast cancer are recommended for chemotherapy, preferably in the neoadjuvant setting. Beyond advantages such as a better operability and an improved assessment of individual prognosis, the preoperative administration of systemic treatment offers the unique possibility of selecting postoperative therapies according to tumor response. In patients with HER2-positive disease, both the escalation of therapy in the case of high-risk features and the de-escalation in patients with a low tumor load are currently discussed. Patients with small node-negative tumors receive primary surgery and, upon confirmation of pathological T1 N0 status, de-escalated adjuvant therapy with paclitaxel and trastuzumab. For those with a large tumor and/or nodal involvement, neoadjuvant polychemotherapy with a dual antibody blockade is recommended. Patients with invasive residual disease benefit from switching postoperative therapy to the antibody-drug-conjugate trastuzumab emtansine (T-DM1). In this review, we discuss current evidence and controversies regarding post-neoadjuvant treatment strategies in HER2-positive breast cancer.

## 1. Introduction

The current standard of care for HER2-positive breast cancer (BC) is a combination of chemotherapy with anti-HER2 treatment and patients with a high-risk situation should receive preoperative systemic therapy. In the neoadjuvant setting, chemotherapy is usually combined with two monoclonal antibodies, trastuzumab and pertuzumab, both in a node-negative and a node-positive setting, and anti-HER2 agents are administered synchronously to taxanes. In a low-risk situation (i.e., tumor size ≤2 cm and negative lymph nodes), primary surgery without neoadjuvant therapy should be considered to allow the de-escalation of adjuvant systemic treatment to monochemotherapy with 12 cycles of paclitaxel weekly combined with trastuzumab for one year, as in the APT trial [1,2]. In case of a pathological tumor size over 2 cm, standard adjuvant polychemotherapy with trastuzumab is recommended and patients with clinically unsuspicious nodes (cN0), but with pathological nodal involvement (pN+), should receive both trastuzumab and pertuzumab, based on the APHINITY trial [3]. In this phase III trial, 4805 HER2-positive BC patients (3005 node-positive and 1799 node-negative) treated with adjuvant chemotherapy were randomized 1:1 to anti-HER2 treatment with either trastuzumab and pertuzumab or trastuzumab and a placebo for one year. The majority of patients received anthracycline-containing chemotherapy regimens (77.7% in the pertuzumab arm and 78.1% in the placebo arm, respectively). The 6-year invasive disease-free survival (iDFS) was significantly improved in node-positive patients receiving dual antibody therapy, compared with the placebo group (87.9% vs. 83.4%, hazard ratio (HR) 0.72, 95% CI; 0.59 to 0.87), but not in the node-negative cohort (95% vs. 94.9%, HR 1.02, 95% CI 0.69–1.53). There was no overall survival (OS) benefit observed in the pertuzumab arm 6 years after randomization (95% vs. 94%, HR 0.85, 95% CI, 0.67 to 1.07; *p* = 0.17); however, a longer follow-up is needed to fully assess the OS benefit in the Aphinity trial.

Regarding neoadjuvant chemotherapy, several studies in the last years have compared anthracycline-containing versus anthracycline-free regimens in HER2-positive BC, showing non-inferiority of anthracycline free-treatment schedules in terms of pCR rates and survival. Therefore, especially in consideration of long-term cardiotoxicity, anthracycline-free regimens should be considered in this population [1,4]. The major randomized trials on neoadjuvant therapy in HER2-positive non-metastatic breast cancer are summarized in Table 1.

## 2. Association of Therapy Response with Prognosis and the Concept of Post-Neoadjuvant Treatment

**Figure 1:**
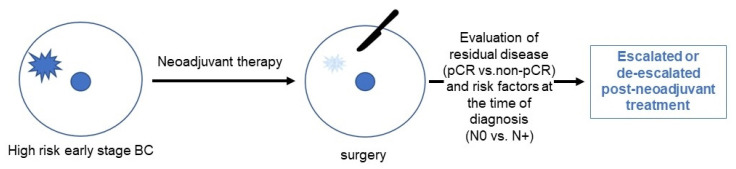
A flow of neoadjuvant and post-neoadjuvant treatment in breast cancer.

Neoadjuvant therapy (NAT) in BC offers several advantages, such as improving chances for breast-conservation due to tumor shrinkage and a better assessment of individual prognosis. Since the pathological response to NAT is considered a valid surrogate parameter for long-term survival in BC patients, the pathological complete response (pCR) is commonly used as an endpoint in neoadjuvant trials [12,13]. The pooled analysis of 9440 BC patients from 14 neoadjuvant studies by Cortazar et al. confirmed pCR as a strong independent prognostic factor with the highest level of evidence [14]. Patients who achieved pCR, defined as ypT0/Tis ypN0, had significantly better event-free survival (EFS; HR 0.48, 95% CI 0.43–0.54) and OS (HR 0.36, 95% CI 0.31–0.42) than those with non-pCR. This association was particularly strong in patients with triple-negative and HER2-positive BC. However, patients did not benefit from administration of neoadjuvant therapy in terms of relapse risk reduction [15]. Therefore, beyond treatment strategies developed to improve pCR rates, an approach of additional postoperative treatment for high-risk patients that did not achieve pCR, so-called post-neoadjuvant therapy (Figure 1), has become a major research focus in recent years. Following the publication of results from first randomized post-neoadjuvant trials such as KATHERINE (HER2-positive BC) and CREATE-X (HER2-negative BC), a post-neoadjuvant therapy has been incorporated into national and international guidelines [1,4,16]. In this review of the literature, we discuss current evidence and controversies regarding post-neoadjuvant escalation and de-escalation strategies in HER2-positive breast cancer. We searched digital libraries for keywords related to “neoadjuvant therapy”, “HER2-positive breast cancer”, and “post-neoadjuvant therapy”.

## 3. Post-Neoadjuvant Treatment in HER2-Positive Breast Cancer Depending on Response to Primary Therapy

### 3.1. Post-Neoadjuvant Therapy in Patients with Non-pCR

In patients who did not achieve pCR following neoadjuvant therapy, the postoperative treatment should be switched to T-DM1 with 14 courses of the antibody-drug-conjugate administered in the post-neoadjuvant setting [1,4,16]. This recommendation is based on the results of the KATHERINE trial [17]. In this multicenter, open-label, phase three trial, 1486 HER2-positive patients (cT1-cT4 cN0-cN3 M0; cT1a/b cN0 excluded) with residual invasive tumors after neoadjuvant treatment were randomized 1:1 to post-neoadjuvant therapy with trastuzumab alone versus T-DM1. Patients had to receive at least six cycles (16 weeks) of conventional neoadjuvant chemotherapy containing a minimum of 9 weeks taxane-based treatment and 9 weeks of trastuzumab. The administration of anthracyclines was not required in the study protocol, however, 75.9% of patients in the trastuzumab arm and 77.9% in the T-DM1 arm received neoadjuvant anthracycline. Regarding anti-HER2 therapy, most patients were treated with trastuzumab alone (80.2% in standard group and 80.8% in T-DM1 group, respectively), whereas 18.7% patients in the standard arm and 17.9% in the T-DM1 arm received the two antibodies trastuzumab and pertuzumab in the neoadjuvant setting. In patients with HR-positive HER2-positive disease, a concomitant administration of endocrine therapy and anti-HER treatment was allowed by the study protocol. Patients treated with T-DM1 had a significantly improved 3-year iDFS compared to those receiving trastuzumab (88.3% vs. 77.0%, respectively, HR 0.50, 95% CI, 0.39–0.64; *p* < 0.001). The distant recurrence risk was also lower in patients who received T-DM1 compared to those treated with trastuzumab (3-year freedom from distant recurrence 89.7% vs. 83%, respectively, HR 0.60, 95% CI, 0.45–0.79). No difference in overall survival between both groups has been demonstrated so far.

### 3.2. Neratinib as a Post-Neoadjuvant Treatment Option in HR-Positive HER2-Positive Patients

In patients with HR-positive HER2-positive tumors, after the completion of trastuzumab-based treatment for 1 year, a therapy-escalation with an orally available tyrosine kinase inhibitor neratinib for another year can be considered based on data from the ExteNET trial [18]. Neratinib binds to and irreversibly inhibits the HER2 receptor tyrosine kinase, thereby reducing autophosphorylation in cells and inhibiting downstream signals and cell regulatory pathways resulting in a decreased cellular proliferation. In this phase III multicenter study, 2480 HER2-positive patients with stage 1–3c disease (1–3c in original protocol, modified to stage 2–3c in February, 2010) after one year of treatment with trastuzumab were randomized to post-(neo)adjuvant treatment with neratinib 240 mg/d versus placebo for another year. Patients treated with neratinib showed a significantly improved 5-year-DFS compared to the placebo arm (90.2% vs. 87.7%, HR 0.73, 95% CI, 0.57–0.92; *p* = 0.0083). However, this effect has been demonstrated only in the HR+ subgroup of the study population. The most common adverse event of grade three or higher was diarrhea, developed in 40% of the neratinib-treated patients. Therefore, prophylaxis with loperamide is recommended during treatment with this tyrokinase inhibitor. Furthermore, a dose escalation strategy at the beginning of the therapy (escalation from 120 to 240 mg over 2 weeks) has shown to reduce diarrhea significantly [19].

Since patients who achieved pCR after neoadjuvant therapy have been excluded from the ExteNET study, neratinib should only be considered in patients treated in an adjuvant setting or patients with a residual tumor after neoadjuvant treatment [20]. The interpretation of these results is challenging for several reasons. All patients in the ExteNET trial received trastuzumab only before randomization, whereas most HER2-positive patients receive trastuzumab and pertuzumab nowadays and switch to T-DM1 in case of non-pCR. The efficacy of neratinib after T-DM1 has not been investigated so far. In the context of neoadjuvant and post-neoadjuvant strategies, the question of the therapeutic relevance of neratinib in the current clinical landscape remains therefore to be clarified.

### 3.3. Future Perspective: Trastuzumab Deruxtecan

Another promising option for post-neoadjuvant treatment is the antibody-drug-conjugate trastuzumab deruxtecan (T-DXd). In the DESTINY-Breast03 trial including 524 patients with HER2-positive metastatic BC pretreated with trastuzumab and taxane, T-DXd as second-line therapy was compared against the current standard T-DM1. Patients who received T-DXd showed a significantly longer progression-free survival (PFS) than those treated with T-DM1 (25.1 versus 7.2 months, respectively, HR = 0.2649, 95% CI, 0.2011–0.3489; *p* = 6.5 × 10^−24^) [21]. Based on these results, post-neoadjuvant therapy with T-DXd versus T-DM1 in patients with non-pCR is currently being investigated within the phase III Destiny-Breast05 (TRUDY) trial (NCT04622319).

### 3.4. Post-Neoadjuvant Therapy in Patients with pCR

In patients achieving pCR, current guidelines recommend the continuation of trastuzumab +/− pertuzumab to complete one year of treatment [4,22]. For those initially presenting with node-positive disease, pertuzumab should be considered based on the results of the adjuvant APHINITY trial [3], but the actual additional benefit of continuing anti-HER2 therapy beyond surgery in this setting remains unclear, since all patients in the APHINITY trial received chemotherapy and anti-HER2 therapy in the adjuvant setting. As in patients with non-pCR, a concomitant administration of endocrine therapy and anti-HER therapy is recommended by national and international guidelines [1,4].

## 4. Treatment De-Escalation in Selected Patients

### 4.1. HER2-Positive HR-Negative Disease

The WSG-ADAPT HER2+ HR- trial examined outcomes after de-escalated neoadjuvant treatment in 134 patients with HR-negative HER2-positive disease [23,24]. Most patients presented with tumors ≤5 cm (40% cT1, 52% cT2) and 57% were clinically node-negative. In the neoadjuvant setting, patients received 12 weeks of a chemotherapy-free regimen (trastuzumab/pertuzumab) or paclitaxel weekly combined with trastuzumab/pertuzumab. After surgery, therapy according to national standards was recommended, i.e., four cycles of epirubicin/cyclophophamide for all patients and in addition 12 × paclitaxel weekly for those in the neoadjuvant chemotherapy-free arm. The trial showed exceptionally high pCR rates in the de-escalated chemotherapy arm, with 90.5% of patients achieving ypT0/is ypN0 after 12 weeks of the paclitaxel + dual antibody blockade [24]. pCR rates were substantially lower in the chemotherapy-free arm. Importantly, chemotherapy after surgery could be omitted for patients achieving pCR at the investigator’s discretion and 29% of patients who achieved pCR in the chemotherapy-free arm as well as 79% in the paclitaxel-arm received no further post-neoadjuvant chemotherapy [23]. At the 2021 ASCO Annual Meeting, Harbeck et al. presented the first survival results of the trial. All relevant endpoints (5-year-iDFS, dDFS, and OS) after 5 years were lower in patients receiving the chemotherapy-free regimen in the neoadjuvant setting. Interestingly, the 5-year-iDFS was similar in patients receiving neoadjuvant paclitaxel + dual antibody therapy and in those achieving pCR in the chemotherapy-free arm (both 98%, respectively), suggesting that chemotherapy-free regimens might be an option in selected patients. To further examine this issue, intrinsic subtypes were evaluated, with most tumors (72.4%) being HER2-enriched. The pCR rates after the chemotherapy-free neoadjuvant treatment were higher in patients with non-basal subtypes, while no patient with a basal tumor reached pCR. Another tool bearing potential to identify patients with an excellent prognosis after chemotherapy-free treatment might be the KI67-based early assessment of response. In the WSG-ADAPT HER2+ HR- trial, patients received a second minimally invasive biopsy after 3 weeks of treatment. The early-response criterion was defined as either a relative Ki67 decrease of at least 30% compared to baseline (“proliferation response”) or <500 invasive tumor cells (“cellularity response”). Interestingly, in the chemotherapy-free arm, 49% of early responders with HER2 3+ tumors achieved pCR, defined as no invasive residual tumor (ypT0/is ypN0), and the authors concluded that chemotherapy-free regimens might be a promising option in a highly selected population and that future research should focus on identifying patients most likely to be able to forego chemotherapy [23].

### 4.2. Her2-Positive HR-Positive Disease

Another trial from the WSG study group, the ADAPT-TP HER2+/HR+ study, focused on patients with ER- and/or PR-positive disease [25,26]. This trial analyzed pCR rates in 375 patients with stage I-IV (including only four patients with stage IV disease) HER2+ HR+ BC treated with neoadjuvant T-DM1 + endocrine therapy (ET) vs. trastuzumab + endocrine therapy vs. T-DM1 alone for 12 weeks [26]. pCR, defined as ypT0/is ypN0, was achieved by 41% of patients treated with T-DM1, 41.5% of patients treated with T-DM1 + ET, and only 15.1% with trastuzumab and ET (*p* < 0.001). All patients received post-neoadjuvant chemotherapy according to national standards (4 EC +/− 12 × paclitaxel + trastuzumab for 40 weeks). In patients with pCR, after 12 weeks of therapy a post-neoadjuvant chemotherapy could be omitted. Recently, an association of immune markers and tumor-related biomarkers and a response to de-escalated treatment has been reported [25]. Interestingly, pCR was lower in PIK3CA-mutated tumors compared with the wildtype, and the HER2-enriched subtype was associated with an increased pCR rate in both T-DM1 arms (54% vs. 28%). After a median follow-up of 5 years, no significant differences between arms were observed regarding DFS (T-DM1/T-DM1+ET/T+ET 5-y rate: 88.9%, 85.3%, and 84.6%) and OS (97.2%, 96.4%, and 96.3%). pCR was associated with improved DFS (5y DFS 92.7% vs. 82.7, HR = 0.40, 95% CI 0.18–0.85). A total of 41 out of 117 patients with pCR received no further chemotherapy and a similar 5-year-DFS was observed irrespective of chemotherapy administration (pCR and adjuvant chemotherapy: 92.1% (95%-CI: 78–97%) vs. pCR and not adjuvant chemotherapy: 93% (84–97%)).

In the phase II WSG-TP-II trial, 207 patients with HER2-positive/HR-positive breast cancer were randomized to 12 weeks of therapy with paclitaxel weekly + trastuzumab + pertuzumab (*n* = 107) vs. 12 weeks of endocrine therapy (tamoxifen or aromatase inhibitor) + trastuzumab + pertuzumab, achieving pCR rates of 57% and 24%, respectively [27]. All patients received trastuzumab and pertuzumab in a post-neoadjuvant setting for one year, an omission of adjuvant chemotherapy was allowed in patients achieving pCR. The survival results of this trial are currently awaited.

### 4.3. Her2-Positive Disease Regardless of HR-Status

Another study investigating treatment de-escalation was the KRISTINE trial [28]. In this phase III study, 444 patients with stage II-III HER2-positive breast cancer were randomized to neoadjuvant polychemotherapy (TCH+P) vs. a combination of T-DM1 and pertuzumab. Patients receiving TCH+P were treated with trastuzumab and pertuzumab after surgery and those in the T-DM1 + pertuzumab arm continued the same therapy postoperatively. De-escalated therapy led to a lower pCR rate, compared to TCH+P (44.4% vs. 55.7%, *p* = 0.016). After a median follow-up of 37 months, the risk of an EFS event was higher with T-DM1 and pertuzumab (HR 2.61 (95% CI, 1.36 to 4.98)). Particularly, more locoregional progression events were observed before surgery (6.7% vs. 0% in the TCH+P arm). The risk of an iDFS event after surgery was similar between arms.

Whether the imaging-based early evaluation of responses may select patients more likely to achieve pCR through de-escalated neoadjuvant therapy was investigated in the TBCRC026 trial [29,30]. In this single-arm study, the response to a chemotherapy-free regimen (12 weeks of trastuzumab plus pertuzumab) was assessed early (i.e., 15 days after the start of treatment) using PET-CT. Early changes predicted a response, but authors concluded that this quantitative imaging strategy should be further optimized. Whether the evaluation of intrinsic subtypes may further contribute to an optimized selection of candidates for therapy de-escalation remains to be clarified [31]. Further trials addressing therapy de-escalation in the neoadjuvant setting are summarized in Table 2.

The main critical issue regarding de-escalated neoadjuvant treatment is the question of the optimal post-neoadjuvant strategy to be offered in case of non-pCR. Current AGO Breast Committee guidelines recommend primary surgery in patients with low-risk HER2-positive disease (i.e., cT1 cN0), followed by de-escalated adjuvant treatment with 12 cycles of weekly paclitaxel and one year of trastuzumab in those who remain as low-risk upon postoperative pathological evaluation (pT1 pN0) [1]. According to NCCN guidelines, neoadjuvant paclitaxel and trastuzumab may be considered in a low-risk setting, particularly in patients not eligible for other standard adjuvant regimens due to comorbidities [4]. However, the question arises whether patients still harboring invasive residual disease following this de-escalated regimen would have reached pCR through standard-dosed neoadjuvant therapy. Further, this setting has not been investigated in the KATHERINE trial that enrolled patients with a residual tumor after at least 16 weeks of conventional preoperative chemotherapy. Therefore, it remains unclear whether patients with non-pCR following weekly paclitaxel in combination with anti-HER2 therapy should be recommended T-DM1 in the post-neoadjuvant setting. Hypothetically, completing anthracycline/cyclophosphamide or carboplatin might also be an option, but all these strategies are based on expert opinion rather than evidence from clinical trials. Therefore, data from ongoing clinical studies such as CompassHER2pCR (NCT04266249) or DECRESCENDO (NCT04675827) addressing this issue have to be awaited (Table 2).

## 5. Conclusions

Patients with high-risk HER2-positive breast cancer benefit from neoadjuvant chemotherapy in combination with anti-HER2 treatment. Beyond advantages such as a better operability due to the reduction of tumor mass and the improved assessment of prognosis based on response to therapy, the main reason for administering therapy in a neoadjuvant setting is the possibility of selecting patients in need for post-neoadjuvant strategies. Patients with residual invasive cancer benefit from switching anti-HER2 therapy to T-DM1 instead of receiving the monoclonal antibodies trastuzumab +/− pertuzumab. On the other hand, those achieving pCR may be recommended the de-escalation of therapy. The optimal post-neoadjuvant therapy for patients with residual invasive tumors following de-escalated neoadjuvant therapy currently remains a matter of debate. Therefore, some guidelines discourage the use of de-escalated regimens (e.g., paclitaxel plus anti-HER2 therapy) in the neoadjuvant setting [1].

## Figures and Tables

**Table 1:** The major clinical studies on neoadjuvant therapy in HER2-positive non-metastatic breast cancer (only randomized phase II and III trials).

Trial	Patient Number and Setting	Treatment Arms	pCR Rate	Survival	Post-Neoadjuvant Therapy
TRAIN-2 [5,6] Randomized, phase III	438 Stage II-III	2 arms: 3 × FEC (500/90/500 mg/m^2^ q3w, followed by 6 × paclitaxel 80 mg/m^2^ day 1, 8 + carboplatin AUC 6 q3w (or AUC 3 day 1, 8) vs. 9 × paclitaxel/carboplatin); + trastuzumab/pertuzumab in both arms	67% vs. 68% ^(1)^	3-y-EFS: 92.7% vs. 93.6% 3-y-OS: 97.7% vs. 98.2%	Trastuzumab to complete 1 year of treatment in both arms
TRYPHAENA [7,8] Randomized Phase II	225 T2-3 N0-3 or T4 Tumor size > 2 cm	3 arms: (A) 3 × FEC + trastuzumab/pertuzumab q3w → 3 × docetaxel + trastuzumab/pertuzumab (B) 3 × FEC → 3 × docetaxel + trastuzumab/pertuzumab (C) 6 × docetaxel + carboplatin + trastuzumab/pertuzumab	61.6% vs. 57.3% vs. 66.2% ^(1)^ 50.7% vs. 45.3% vs. 51.9% ^(2)^	3-y-DFS: 87% vs. 88% vs. 90% 3-y-PFS: 89% vs. 89% vs. 87%	Treatment according to local guidelines
TRIO-US B07 [9] Randomized Phase II	128 Stage I-III	3 arms: 6 × carboplatin/docetaxel q3w + (A) trastuzumab (B) lapatinib (C) trastuzumab + lapatinib	47% vs. 25% vs. 52% ^(1)^	NR	Treatment according to local guidelines
NSABP B-52 [10] Randomized Phase III	315 HER2+ HR+ N+ or tumor size ≥ 2 cm	2 arms (A) 6x docetaxel/carboplatin/trastuzumab, and pertuzumab q3w (B) 6x docetaxel/carboplatin/trastuzumab, and pertuzumab q3w + endocrine therapy ^(3)^	40.9% vs. 46.1%, respectively, (*p* = 0.36) ^(1)^	NR	Treatment according to local guidelines
NeoSphere [11] Randomized phase II	417 T2-4, N0-3 Tumor size ≥ 2 cm	4 arms: (A) 4x trastuzumab + docetaxel (B) 4x trastuzumab + pertuzumab + docetaxel (C) 4x trastuzumab + pertuzumab (D) 4x pertuzumab + docetaxel	31% vs. 49% vs. 18% vs. 23%	5-y-DFS 81% vs. 84% vs. 80% vs. 75%	Trastuzumab for 1 year + completion of chemotherapy (group A, B, D 3 × FEC Group C: 4x DOC + 3 × FEC)

^(1)^ Defined as ypT0/is ypN0. ^(2)^ Defined as ypT0 ypN0. ^(3)^ Aromatase inhibitor for postmenopausal and an aromatase inhibitor plus ovarian suppression in premenopausal patients. Abbreviations: NR—not reported, FEC—fluorouracil, epirubicin, cyclophosphamide, AUC—area under the curve, EFS—event-free survival, DFS—disease-free survival, OS—overall survival, PFS—progression-free survival.

**Table 2:** Clinical studies investigating treatment de-escalation in HER2-positive non-metastatic breast cancer.

Trial	Patient Number and Setting	Treatment Arms	pCR Rate	Survival	Post-Neoadjuvant Therapy
WSG-ADAPT HER2+/HR- [23,24,32], randomized, phase II	134 ER and PR- cT1-4c	2 neoadjuvant arms: 4 × trastuzumab + pertuzumab q3w without chemotherapy vs. 4 × trastuzumab + pertuzumab q3w + 12 × paclitaxel 80 mg/m^2^ weekly	34.4% vs. 90.5% ^1)^ 24.4% vs. 78.6% ^(2)^	5-y-iDFS: 87% vs. 98% 5-y-dDFS: 92% vs. 98% 5-y-OS: 94% vs. 98%	40 weeks trastuzumab + completion of chemotherapy (either EC in neoadjuvant paclitaxel arm or EC/P in chemotherapy-free arm); in pts. with pCR chemotherapy could be omitted at the investigator’s discretion
WSG-ADAPT-TP HER2+/HR+ [25]; randomized, phase II	375 ER and/or PR+ cT1-4c	3 neoadjuvant arms: 12 weeks T-DM1 vs. 12 weeks T-DM1 + endocrine therapy vs. 12 weeks trastuzumab + endocrine therapy	41% vs. 41.5% vs. 15.1% ^(1)^	5-y-DFS: 88.9% vs. 85.3% vs. 84.6%) 5-y-OS: 97.2% vs. 96.4% vs. 96.3	4 × EC in all patients, followed by 12 weeks of paclitaxel weekly (in patients treated with trastuzumab and endocrine therapy), trastuzumab for 40 weeks; in case of pCR at surgery, additional chemotherapy could be omitted at the discretion of the investigator
WSG TP II HER2 +/HR+ [27] randomized, phase II	207 HER2+/ HR+	2 neoadjuvant arms 12 weeks paclitaxel weekly + trastuzumab + pertuzumab q3w vs. 12 weeks of endocrine therapy + trastuzumab + pertzumab q3w	57% vs. 24% ^(1)^	NR	Standard of care; Trastuzumab and pertuzumab for 1 year in all patients. Omission of further chemotherapy was allowed in all patients with pCR;
KRISTINE [28,33], randomized, phase III	444 Stage II-III and tumor size > 2 cm	2 neoadjuvant arms: 6 × T-DM1 + pertuzumab q3w vs. 6 × docetaxel + carboplatin + trastuzumab/pertuzumab q3w	44% vs. 56% ^(1)^	3-y-EFS: 85.3% vs. 94.2% (HR for EFS: 2.61) 3-y-iDFS: 93.0% vs. 92.0% (HR for iDFS: 1.11) 3-y-OS: 97.0% vs. 97.6% (HR for OS: 1.21)	Continuation of HER2-targeted treatment every 3 weeks for a total of 18 cycles-inclusive of neoadjuvant and adjuvant therapy
TBCRC023 [34]; randomized, phase II	97 Tumor size ≥ 2 cm	2 neoadjuvant arms: 12 weeks of lapatinib + trastuzumab weekly vs. 24 weeks of lapatinib + trastuzumab weekly Pts. with ER and/or PR-positive tumors received additional letrozole daily +/− LHRHa	12% vs.28% ^(1)^ ER-positive: 9% vs. 33% ER-negative: 20% vs. 18% ^(1)^	NR	At the discretion of the treating physician, details not reported
TBCRC026 [29,30], single arm, phase II	88 Stage II-III ER-	4 cycles of neoadjuvant trastuzumab/pertuzumab	22%	NR	Recommended “per standard of care”, details not reported
PAMELA [31], single arm, phase II	151 Stage I-IIIA	18 weeks of lapatinib + trastuzumab Pts. with HR-positive tumors received additional endocrine therapy	30%	NR	According to the physician’s discretion, details not reported
CompassHER2PCR, NCT04266249, single arm, phase II, ongoing	Estimated enrollment 2156 stage II-IIIa	4 cycles of taxane (Paclitaxel weekly or Docetaxel or nab-Paclitaxel) + trastuzumab + pertuzumab q3w			13 cycles of trastuzumab+pertuzumab q3w in patients with pCR ^(1)^; 14 cycles of T-DM1 in patients with non-pCR. An additional standard of care chemotherapy is allowed as well as endocrine therapy, if appropriate
DECRESCENDO NCT04675827, single arm, phase II, ongoing	Estimated enrollment 1065 HER2+ HR- patients Tumor size 15–50 mm, N0	12 weeks of taxane (paclitaxel weekly or Docetaxel q3w) + 4 cycles of subcutaneous pertuzumab/trastuzumab q3w			14 cycles of subcutaneous pertuzumab/trasuzumab in patients with pCR ^(1)^, 14 cycles of T-DM1 in patients with non-pCR. 3–4 cycles of anthracycline based chemotherapy may be administrated before T-DM1 in patients with Residual Cancer Burden score ≥2 at investigator’s discretion.

^(1)^ Defined as ypT0/is ypN0. ^(2)^ Defined as ypT0 ypN0. Abbreviations: NR—not reported, pCR—pathological complete response, EFS—event-free survival, DFS—disease-free survival, iDFS—invasive disease-free survival, Ddfs—distant disease-free survival, OS—overall survival. ----- trials on HR-/HER2+ disease. ----- trials on HR+/HER2+ disease.
